# Joint Node Selection and Power Allocation for Cooperative Positioning Based on Bidding Auction in VANET

**DOI:** 10.1007/s11036-023-02165-z

**Published:** 2023-07-28

**Authors:** Geng Chen, Lili Cheng, Xiaoxian Kong, Qingtian Zeng, Yu-Dong Zhang

**Affiliations:** 1 College of Electronic and Information Engineering, Shandong University of Science and Technology, Qingdao, 266590 China; 2 School of Computing and Mathematical Sciences, University of Leicester, Leicester, LE1 7RH UK

**Keywords:** Internet of vehicles, Cooperative positioning, Bidding auction, Node selection, Power allocation, Positioning accuracy

## Abstract

Due to buildings blocking GPS and Wi-Fi signals, traditional techniques can’t offer the user’s required positioning accuracy in resource-constrained underground parking, but the cooperation of agent nodes can provide the exact localization information to improve the positioning accuracy. However, some well-localized agents may not be willing to sacrifice additional power to improve the others’ positioning accuracy. To encourage cooperation among nodes and allocate transmission power reasonably, this paper proposes a bidding-auction-based cooperative localization (BACL) algorithm to improve the positioning accuracy of agent nodes by joint node selection incentive and power allocation strategy. Firstly, the contribution of channel parameters and prior localization information of agent nodes for positioning accuracy are quantified and an incentive mechanism of cooperative localization from an economic perspective is proposed. Secondly, a virtual currency incentive rule is developed to compensate agent nodes of cooperative localization reasonably due to the consumption of energy for transmitting their location information. Finally, the simulation results have shown that the proposed BACL algorithm has excellent performance in terms of localization accuracy in resource-constrained scenarios. Compared with the full-power cooperative localization (FPCL) and non-cooperative localization (NCL) algorithms, the proposed BACL algorithm improved the positioning accuracy by 10% and 65%, respectively. Meanwhile, compared with the FPCL algorithm, the proposed algorithm reduced resource consumption by 50%.

## Introduction

With the development of the economy and the Internet of Vehicles(IoV), cars have become an indispensable part of people’s lives and the increase in the number of cars has caused the problem of difficult parking [1, 2]. To solve the problem of difficult parking, governments and departments at all levels increase the investment in transportation infrastructure every year to build, expand and extend the scale of underground parking lots [3]. However, due to buildings blocking the Global Positioning System (GPS) and Wireless Fidelity (Wi-Fi) signals in underground parking, it is difficult to achieve accurate positioning using traditional technology in the urban environment [4]. The United States Department of Transportation (USDOT) and other government agencies around the world are committed to using vehicle ad hoc network(VANET) technology to improve transportation safety, mobility, and anti-interference [5, 6]. VANET is a promising example of providing high-precision location information in GPS challenge scenarios [7]. In VANET, there are two types of nodes, called anchor nodes and agent nodes. The former has exact location information, while the latter has no exact location information but can obtain initial location information through the anchor nodes [8]. We use connected-vehicle (CV) technology in VANET, this technology is characterized by information sharing between vehicles (V2V), between vehicles and infrastructure (V2I/I2V), and between other related entities (V2X) [9]. Information is shared through different media, including wireless communication, such as dedicated short-range communication (DSRC). In the underground parking, when we are looking for our vehicles, we sometimes cannot locate the vehicle location due to human reasons. In addition, the blocking of buildings affects the driver’s vision, it is also necessary to give vehicle positioning for sending collision warnings and other safety information in time [10].

Considering the resource limitation of the underground parking and the consumption of too many anchor nodes, and to ensure satisfactory positioning accuracy with reasonable power consumption for vehicles in the underground parking lot, we adopt the cooperative positioning method. However, agent nodes take more resources for range measurement and information exchange for cooperation. Although the cooperative approach can achieve a better positioning performance, it is “unfair” to some already well-localized agents which would not like to sacrifice their power for improving the others’ localization accuracy. It is very important to study how to encourage agent nodes to participate in cooperative localization.

Based on these motivations, we developed a bidding-auction-based node selection and power allocation algorithm in the vehicle location network of the resource-constrained underground parking to solve the problem of location information transmission and resource competition between agent nodes. An incentive rule with virtual currency is developed to give agent nodes sharing location information get compensation reasonably. The more virtual currency the seller gets, he can buy the more location information from other agent nodes in the future. When the auction starts, the agent node with more accurate location information and more residual energy consumes its transmission power to transmit its location information to the buyer. After receiving the bid, the buyer selects the appropriate node as the best cooperation node and checks its remaining virtual currency. After that, the best agent node selected performs range measurement and obtains the corresponding virtual currencies after successful transactions. Simulation results show that the excitation scheme improves positioning performance, saves transmission power, and provides a solution to the positioning problem in resource-constrained environments.

In this paper, we proposed a BACL algorithm to solve the positioning problem of agent vehicles by joint node selection and power allocation strategy. The main contributions of this paper are as follows.

1. Firstly, we consider a novel scenario in resource-constrained underground parking using fixed base stations and mobile vehicles as cooperative localization nodes. In this scenario, the vehicles participate in cooperative localization by rationally distributing the energy of range measurement between them to improve the user’s required localization accuracy.
2. Secondly, the agent node that needs help in positioning acts as the buyer, while other agent nodes that provide collaboration positioning act as the seller, combining node selection incentives of the buyer and power allocation strategies of the seller with solutions to problems posed by vehicle positioning, a game-theoretic equilibrium problem that combines localization accuracy and resource consumption is formulated. An optimal decision is obtained by maximizing the revenue of buyer and seller.
3. Thirdly, the proposed BACL algorithm is used to solve a game-theoretic equilibrium problem with the objective function, and the cooperative node optimally and resource allocation policy by the bidding auction rule is chosen.
4. Finally, we conducted experiments and compared them with algorithms based on the other two methods, which demonstrated the excellent performance of the BACL algorithm in terms of accurate positioning in resource-constrained scenarios, respectively.

The remainder of the paper is organized as follows. Section 2 presents related work, and Section 3 describes the system model and formulates the problem. Section 4 proposed a BACL algorithm to improve vehicle positioning accuracy. Section 5 discusses the simulation parameters and results. Section 6 concludes this work.

## Related work

Location information is attracting more and more attention due to the increasing awareness of its importance for a wide range of applications [11]. Whether it is for tracking valuable assets in the industrial context or monitoring the motion of vehicle to prevent accidents or offering a better driving experience using proximity and positioning marketing, an accurate and efficient indoor positioning has become crucial [12]. Scholars have done a lot of research work on node selection, performance analysis, agent node selection strategy, power allocation methods, etc [13, 14]. Wu et al proposed the system architecture of cooperative positioning and the ultra-wide-band (UWB) multi-source fusion algorithm. The real flight experiment results show that the successful rate of seamless handover is approaching 96% in hybrid complex scenes [15]. Guo et al applied the iterative Bayesian focusing algorithm to the inhomogeneous propagation medium of the sound source to solve the problem of positioning error caused by the non-uniform propagation medium of the sound source [16]. Chauchat et al. solved the cumulative and current nonlinear optimal navigation problems formed in the iterative process of error problems by using collaborative positioning [17]. Dai et al. proposed a node priority strategy for allocating transmission resources among network nodes, and a computational geometry framework for determining the optimal node priority strategy is developed. Simulation results show that this method can significantly reduce the computational complexity of the priority ranking strategy and improve the accuracy of network location [18].

The above research work assumes that the nodes participating in cooperative localization have enough energy to participate in cooperative localization [19, 20]. However, in the resource-constrained underground parking, most of the sensor nodes of the agent vehicles are powered by batteries [21]. When cooperative localization is required, if the sensor node is often selected as the best agent node, it will soon run out of energy and exit the cooperative localization system, which will lead to the localization accuracy of the entire cooperative localization system becoming lower [22].

To prolong the life of each agent node as much as possible and improve the positioning performance, auction theory is introduced into cooperative positioning to deal with issues related to maintaining their initial positioning information, participating in cooperative positioning, and allocating power to transmit their location information [23]. Considering that the agent nodes in the cooperative positioning network are selfish, virtual currency is used to pay for location information services, and a new incentive scheme is proposed to encourage agent nodes to participate in cooperative localization [24]. Pires et al propose a methodology to perform cooperative localization in robotic swarms while they navigate through the environment to improve their overall localization. Furthermore, it overcomes and has reduced space usage and time complexity compared to a traditional centralized EKF-based method [25]. Chen et al. considered resource allocation using a game algorithm, focused on resource management issues in the power domain and frequency domain, proposed Stackelberg equilibrium and link negotiation equilibrium as solutions for effective link selection and power allocation, and verified that this strategy achieves low mean square error of location estimation with less ranging measurements [26]. In [27], the interactive two-way auction theory is used to allocate transmission power between multiple users and target nodes, effectively strengthening resource sharing and maximizing the interests of each user. For the vehicle localization problem of the underground parking with limited resources, an iterative double auction game is applied to solve the power allocation and location accuracy of agent nodes. A reward policy is a good way to solve the selfish behavior of node cooperation [28]. To obtain the best balance between location accuracy and cooperative power consumption, Ke et al. developed a local altruistic power allocation game, in which each agent node not only considers its localization accuracy and power consumption but also considers the location accuracy of its neighbors. Simulation results show the balance between power consumption and location accuracy [29]. Domestic and foreign scholars have developed various game theory methods to optimize the positioning performance of agent nodes [30–32].

As far as energy efficiency is concerned, many techniques have been proposed in order to prolong the network lifetime. These techniques can be classified into two categories: Duty-Cycling and Data-Driven [33]. The objective behind Duty-Cycling is to keep the sensors at their lowest energy as much as possible [34, 35]. What we have done is reduce energy consumption as much as possible while ensuring positioning accuracy. In this work, we propose a cooperative positioning joint node selection and power allocation algorithm based on a bidding auction in a vehicular network. This algorithm can not only ensure positioning accuracy but also reduce energy consumption. We choose appropriate agent nodes to participate in cooperative localization, and allocate transmission resources reasonably, so as to ensure the positioning accuracy and low energy consumption of the cooperative localization system and provide a solution to the positioning problem in resource-constrained environments.

## System model and problem formulation

### System model

**Fig. 1:**
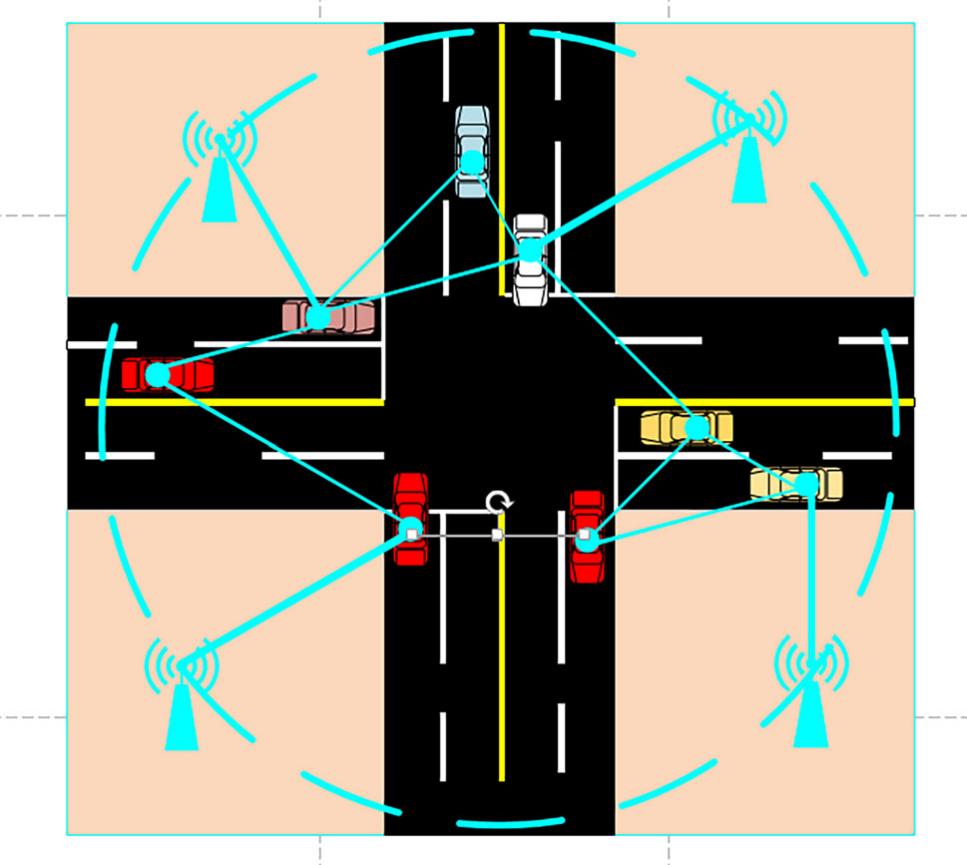
Vehicle-cooperative positioning network

As shown in Fig. 1, this paper considers base stations and vehicles’ cooperative positioning of VANET in underground parking. In the cooperative positioning architecture of VANET, both vehicle terminals and base stations are considered nodes in the network, and the nodes can communicate with each other and make measurements with positioning techniques such as time of arrival (TOA) and received signal strength (RSS) [36, 37]. The vehicle unit can communicate with the base station. The base station acts as an anchor node, i.e., a node with a known absolute position, while the vehicle acts as a mobile node. The vehicle gets the initial location information from the anchor node, and the vehicle can also exchange location information with surrounding vehicles to precise its positioning accuracy. We assume that the network is composed of vehicles and base stations, and the location of base stations are known. The set of vehicles and base stations is defined as follows, $N_c=\{1,2,\dots ,N_c \}$, $N_b=\{1,2,\dots ,N_b\}$. The target node we denote by *k* and the other agent vehicles other than node *k* is denoted by *l*. The angle information denotes $\phi _{kl}$, distance information denotes $d_{kl}$. The real location information of target node k denotes $Q_k=\left[ {x}_k,{y}_k \right] ^{T}$ and estimated location denotes marked as $\tilde{Q}_k=[\tilde{x}_k,\tilde{y}_k]^T$. The location information of agent node *L* is shown as $Q_L=[{x_{L}},{y_{L}}]^T$. The position information of anchor node I is fixed as $Q_I=[{x_{I}},{y_{I}}]^T$. The power consumption of location information transmission from anchor node I to target node k, and from agent node l to target node k are respectively expressed as: ${g_{I,k}}$,${g_{l,k}}$. The candidate agent nodes participating in collaborative localization denote $S_{A(S_l)}$.

Each agent obtains initial position information from the fixed anchor node, which is expressed as $J^{0}_{L}$. Each agent node has the same initial energy. Agent nodes cooperate to locate. Agent *L* sends location information to target node *k*, but it needs to consume its energy. It is assumed that there is no information loss during transmission, and the channels remain unchanged and independent of each other during transmission. The amount of information about the location of agent node *k* can be denoted by

1 $$\begin{aligned} J_(P_l,P_k)=J_{k}^0+\sum \limits _{l=1,l\ne {k}}^{N_c}p_{lk}\xi _{lk}q_{lk}q^T_{lk} \end{aligned}$$

where $J^{0}_{k}$ denotes the initial position information obtained from the fixed anchor node *I*, which is the base station. So the initial information of agent node can be calculated as

2 $$\begin{aligned} J^{0}_k=\sum \limits _{l=1}^{N_b}p_{lk}\xi _{lk}q_{lk}q^T_{lk} \end{aligned}$$

$p_{lk}$ denotes the power consumption between agent *L* and agent *k*. $\xi _{lk}$ denotes the and channel ranging quality between agent *L* and agent *k*, which is directly related to the transmission power and can be given as

3 $$\begin{aligned} \xi _{lk}=\frac{1}{1+p_{lk}q^T_{lk}J^A_0q_{lk}} \end{aligned}$$

$J^A_0$ denotes the equivalent fisher information matrix (EFIM) for the prior positional knowledge of agent nodes. $q_{lk}$ denotes the angle information set between agent *L* and target node *k*, which is expressed as

4 $$\begin{aligned} q_{lk}=\left[ \cos {\Phi {lk}},\sin {\Phi {lk}}\right] ^T \end{aligned}$$

Where $\Phi {lk}$ represents the angle between agent *L* and target node *k*. Due to we derive square position error bound (SPEB) by applying the concept of EFIM to describe positioning accuracy, we can formulate the initial position accuracy of the agent node as

5 $$\begin{aligned} SPEB\_0=tr\left[ \left( J^{0}_k \right) ^{-1} \right] \end{aligned}$$

The minimum transmission location information required by the system is defined as $J_{th}$. If an agent node wants to successfully transmit location information, it must meet $J_(p_l,p_k)\ge {J_{th}}$ , and by combining formula (1), the minimum transmission positioning information must satisfy the following condition

6 $$\begin{aligned} J_{th}\le {J^A_0+\sum _{l=1,l\ne {k}}^{N}g_{lk}\left( m_{lk},m_{kl}\right) U_{kl}U^{T}_{lk} } \end{aligned}$$

Where the $g_{lk}{(m_{lk},m_{kl})}$ denotes the intensity of the ranging information from anchor nodes and the direction of ranging information is denoted by $U_{kl}$.

### Problem formulation

In an energy-constrained wireless sensor cooperative localization system for vehicular networking, each agent node has a limited amount of energy to undertake the task of maintaining the initial location information it acquires and transmitting its location information to other agent nodes. If cooperates with other nodes too often, the energy to transmit its location information to other agent vehicles will be rapidly used up. In addition, if each node is an egotist and retains its energy information without assisting other nodes, the transmission performance of the entire cooperative positioning network will be reduced. Therefore, for better collaborative targeting, two important questions must be addressed. 1) How to select the best agent node to participate in collaborative localization? 2) How much power to allocate for the best agent node to transmit location information? To encourage agent nodes to participate in the collaboration, a virtual currency is introduced to reward agent nodes in collaborative positioning. The more location information the target node intends to obtain through auction to improve its positioning accuracy, the more virtual money it needs to pay. If its virtual currency is insufficient, it cannot purchase more location information and its localization accuracy may be reduced. For agent nodes, assigning more power to the target node in cooperative localization can acquire more virtual currency. However, the transmission power of the agent node to maintain its location information is small, so its positioning accuracy becoming lower. The utility function and expression meaning of its gains and losses are as follows.

1. Target node/buyer: Target node *k* can be modeled as a buyer to obtain the maximum benefit. The utility function of the target node *k* can be defined as.
  7 $$\begin{aligned} U\left( k\right) =tr\left[ P_L\xi _{lk}q_{lk}q^T_{lk}\right] -\pi ^{*}_{L}{*}P_L \end{aligned}$$
  where $\pi ^{*}_{L}$ denotes the final unit power price of agent node *l* and $P_L$ denotes the remaining transmission power. The above income of the target node can be expressed as the difference between the amount of location information obtained from the agent node and the amount of virtual currency paid.
2. Agent node/seller: Each agent node L can be considered as a seller. The agent nodes are designed to earn payments based on the successful transmission of location information. We introduce the concept of power retention rate $\beta _L$ and then measure the remaining transmission power of the agent node by the following Eq. 8.
  8 $$\begin{aligned} P_L=\left( 1-\beta _L\right) P_S \end{aligned}$$

The sellers’ utility function can be defined as the Eq. 9.

9 $$\begin{aligned} U\left( L \right) =tr\left[ \sum _{j=1}^{N_b}X_{lj}\xi _{lj}q_{lk}q^{T}_{lj} \right] +\pi _L\left( 1-\beta _L \right) P_S \end{aligned}$$

where $P_S$ is the unit transmission power and $\pi _L$ is the seller’s optimal price. The utility function of the seller is the sum of the maintenance of their location information and the reward received. Considering the specificity of matrix traces, we let $tr\left[ q_{lj} J^{A}_0q^{T}_{lj}\right] =A$, $tr\left[ q_{lj}q^{T}_{lj} \right] =B$, then the original equation can be simplified to the following Eq. 10.

10 $$\begin{aligned} U\left( L \right) =\frac{\beta _LP_S}{1+\beta _LP_SA}B+\pi _L\left( 1-\beta _L \right) P_S \end{aligned}$$

We simplify the sellers’ optimization function into the following form.

$$\begin{aligned} \mathop {\max } U\left( L \right) \end{aligned}$$

**s.t.**

11 $$\begin{aligned} 0\le {\beta _L}\le {1} \end{aligned}$$

We obtain the following Eq. 12 by taking the second-order derivative of the seller’s utility function.

12 $$\begin{aligned} \frac{\partial ^{2} U\left( L \right) }{\partial ^{2} \beta _L} =\frac{-2P_s}{\left( 1+\beta _LP_sA\right) ^3 } \end{aligned}$$

Where the values of *A*, $P_s$, $\beta _L$ are greater than 0. Therefore, the second-order derivative of the seller’s utility function is greater than 0 and continuous in the domain of definition, in which the function is concave. In optimization theory, the maximum likelihood function can find the maximum value, so the function has a globally optimal solution. In these two utility functions, the power price of each candidate agent node is determined by its state information. Both the buyer and the seller should calculate their maximum profit based on the acquired information, and the final choice of the agent node is determined by the target node.

## Cooperative localization based on bidding auction

There are two basic elements in auction theory: buyer and seller. The target node k that needs the assistance of agent node L acts as the buyer, and the agent node $l_{Nc}\in {S_{A\left( Nc\right) }}$, $N_c=\{1,2,\dots ,N_c \}$ other than target node k is considered as the seller. We use virtual currency to reward the best agent nodes involved in collaborative positioning when solving problems. If a node can obtain more virtual currency, it can purchase more location information from other agents and thus improve its positioning accuracy. During the auction process, agent nodes auction their information by consuming the remaining energy to obtain virtual currency.

### Sellers bidding price

For each agent node, the same amount of virtual currency is initialized in the cooperative localization network system, which we denote by *C*. If a node’s remaining currency amount is less than 0, it is not eligible to be a buyer in the auction system. The agent node submits its node information to the target node, including the remaining energy $E_{rest}$, the remaining funds $C_{rest}$, the contained location information $J^A_0$, and the minimum transmission power $P_L$. To facilitate the reader to understand more clearly the process of obtaining the node information of our agent nodes including the initial position information and the initial position accuracy, the pseudo-code of our proposed algorithm is shown in Algorithm 1.

Compared with the existing power allocation and node selection algorithm, algorithm 1 introduces the concept of virtual currency, so that the localization information can be freely traded among nodes according to the remaining virtual currency, which makes the system gets rid of the central controller and the algorithm can be completely distributed.

**Algorithm 1:**
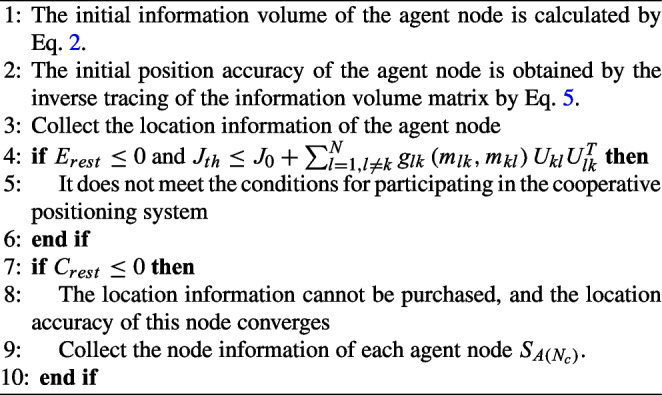
Agent Node Selection under Bidding Auction Algorithm.

According to the information submitted by the agent node, the target node k calculates the demand factor $N_L$, which can be denoted by Formula (13).

13 $$\begin{aligned} N_L=1+exp(-\frac{E_{rest}-C_{rest}}{E_{all}}) \end{aligned}$$

Where $E_{all}$ denotes the initial power of the agent node, we assume that all nodes have the same initial energy. If agent node L has more surplus energy $E_{rest}$ and smaller surplus funds $C_{rest}$, then the demand factor will be smaller. The final price is determined by the optimal price and the demand factor given by each agent node itself. Therefore, the final unit price per unit power of each agent node can be formulated as

14 $$\begin{aligned} \pi ^{*}_L=\pi _LN_L \end{aligned}$$

Equation 14 shows that the final price denote higher than the optimal price, where the price increase is smaller for nodes with more residual energy and larger for nodes with less residual energy. Therefore, we introduce a demand factor that allows the price to respond to the residual energy of the agent nodes. Finally, the cost paid by the target node *k* for the agent node *L* can be presented as follows

15 $$\begin{aligned} C_L=\pi ^{*}_LP_L \end{aligned}$$

### Seller power allocation

In an energy-constrained cooperative positioning system, each agent node wants to retain as much power as possible to maintain its high-precision positioning. Therefore, there is an incentive currency in the collaborative transmission of location information based on bidding auctions, which motivates the agent nodes to earn a certain amount of currency to obtain higher location accuracy later. The maximum power reserved rate for the agent node is expressed using the following equation.

16 $$\begin{aligned} \beta _{th}=\frac{P_S-P_{L\left( th \right) }}{P_S} \end{aligned}$$

Obviously $0\le {\beta _L}\le {1}$. It can be seen from Formula (10) that if the power retention rate of the agent node is smaller, it means that the power to maintain its location information is smaller, but more monetary compensation can be obtained. On the contrary, when $\beta _L$ is large, it means that the power to maintain their location information is large, but the power to participate in the cooperative location transmission of location information will become less, and they will not get more rewards. Therefore, this contradiction can be transformed into a maximization problem, and appropriate $\beta _L$ value can be set to maximize the profit. Based on the above analysis, we take the derivative of the utility function of the agent node, assign its equation to 0, and obtain the following formula.

17 $$\begin{aligned} \frac{\partial U\left( L\right) }{ \partial \beta _L}=tr\left[ P_S\xi _{lj}q_{lj}q^{T}_{lj}\right] -\pi _LP_S \end{aligned}$$

Let $\frac{ \partial {U\left( L\right) } }{ \partial \beta _L}=0$, the expression of $\pi _L$ is as shown in Formula (18).

18 $$\begin{aligned} \pi _L=\frac{B}{\left( 1+\beta _LP_sA\right) ^2 } \end{aligned}$$

Where, $\pi _L$ and $\beta _L$ indicate that when the seller’s profit reaches the maximum, the optimal price and power retention rate are a pair of mapping relationships. The optimal price can be calculated by the power retention rate $\beta _L$. The maximum retention rate of agent nodes in the cooperative positioning system is defined as.

19 $$\begin{aligned} \beta _L=\beta _{th} \end{aligned}$$

Therefore, the following expression can be obtained by introducing formula (18) into formula (10).

20 $$\begin{aligned} U\left( L \right) =\frac{B\beta _LP_S}{1+\beta _LP_SA}+\frac{B}{\left( 1+\beta _LP_SA\right) ^2 }\left( 1-\beta _L\right) P_S \end{aligned}$$

**Fig. 2:**
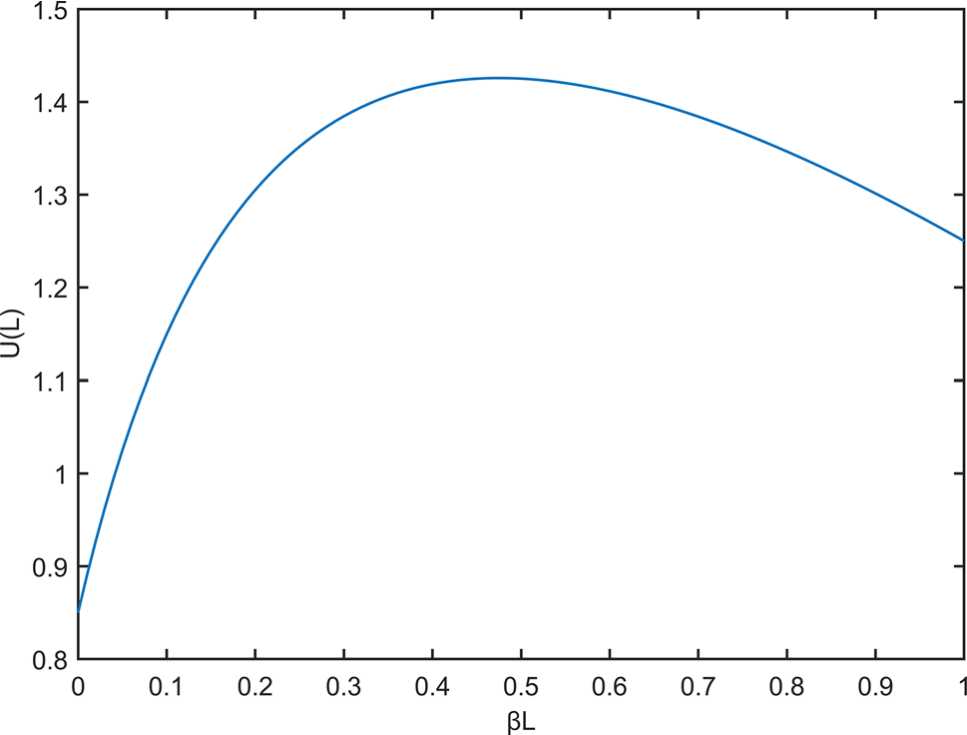
The seller’s earnings function

When the power retention rate meets $\beta ^{*}_L=\beta _{th}$ , the optimal price that maximizes the utility function of the seller can be calculated by Formula (20). The changing trend of the seller utility function with power retention rate is shown in Fig. 2.

### Node selection for buyers

The optimal agent choice depends on the utility function of the destination node, i.e., the buyer. We substitute formulas (8) and (15) into formula (7), which get a set of mapping relationships expressed by the following formula.

21 $$\begin{aligned} l=\arg \max U\left( k,l\right) ,l\in {S_{A\left( S_l\right) } } \end{aligned}$$

The agent node that enables the buyer to obtain the maximum profit is selected as the best node. However, the buyer needs to measure $C_L$ according to their own currency amount before making payment. The buyer’s remaining currency amount $C_{rest}$ must be greater than or equal to $C_L$ . Otherwise, the payment task cannot be completed. In this case, the buyer will send the signal of “insufficient funds” to the best agent node, and it is unnecessary to pay any power cost to send this signal, which means that the buyer is insufficient in funds and the transaction fails.

After the buyer selects the best agent node through the bidding auction algorithm, it will pay the corresponding monetary amount to the best agent node, and the best agent node will transmit its own location information for the buyer. The target node gets the help of the superior agent node, and the selected best agent node will get some monetary compensation during the transaction. In this way, the cooperative positioning of the incentive agent node is completed, and the positioning accuracy of the whole cooperative positioning system is improved, while the power consumption is less. When the virtual currency of target node k is less and there is no surplus amount to pay to the agent node, the auction activity stops.

**Fig. 3:**
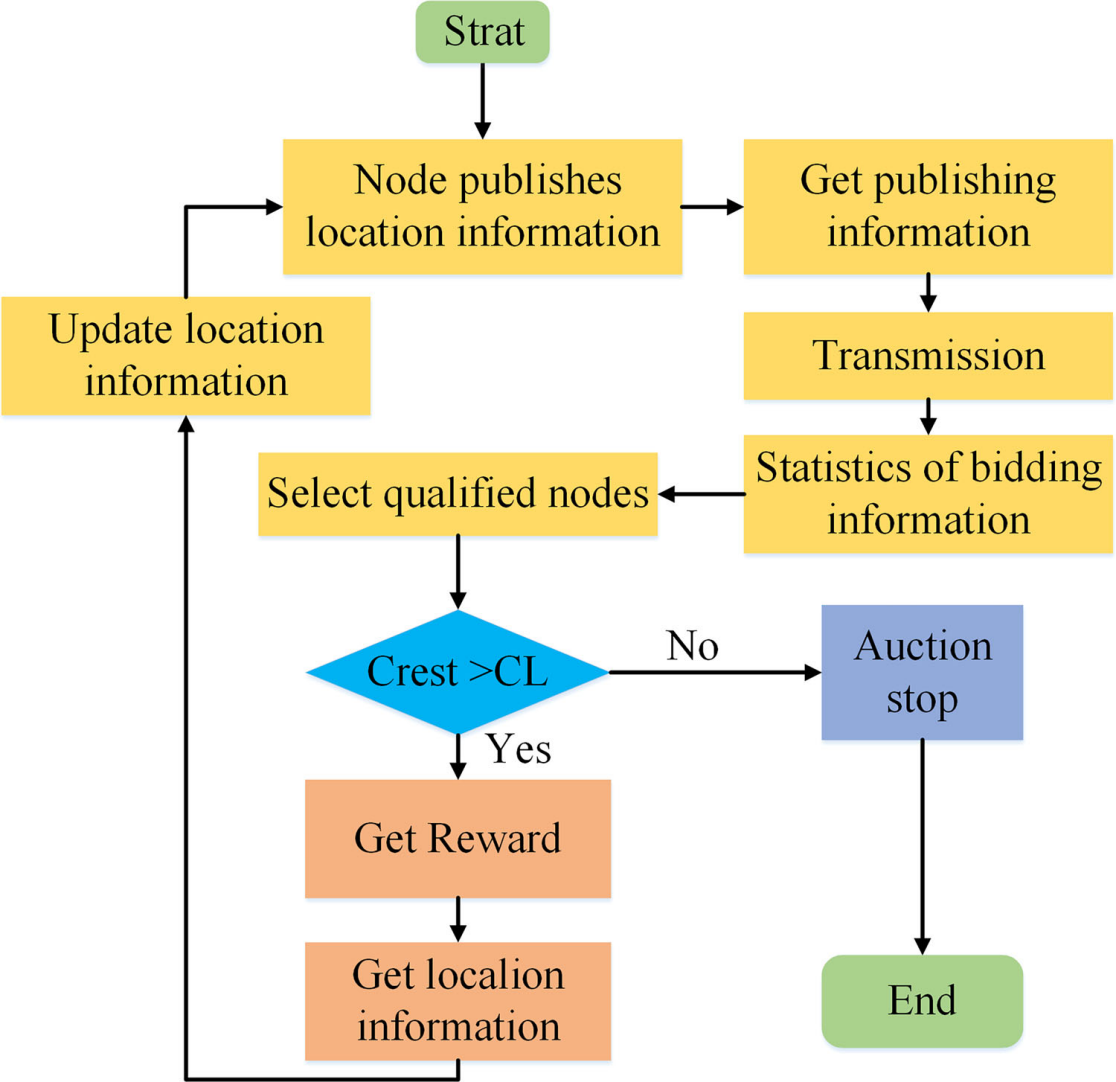
Bidding auction algorithm process

After a predetermined number of iterations, the bidding auction algorithm can obtain the optimal number of nodes participating in collaborative localization and the optimal resource allocation scheme. To help readers understand our process more clearly, the algorithm flow chart is shown in Fig. 3, and the pseudo-code is shown in Algorithm 2.

Compared with the existing algorithms, Algorithm 2 introduces virtual currency into power allocation and node selection, which makes nodes perform node selection and power allocation simultaneously according to the amount of virtual currency, and the algorithm allows the reference nodes to select the optimal target node to participate in the cooperation through iterative bidding auction, thereby improving the energy utilization rate of the system.

**Algorithm 2:**
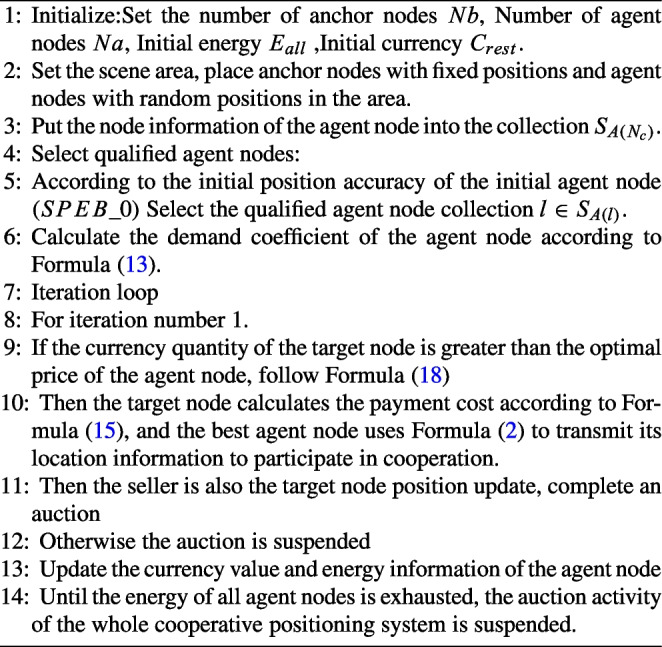
Cooperative positioning and resource allocation algorithm based on bidding auction.

### Performance evaluation criteria

We derive the utility function of the seller above to obtain the optimal power reservation factor. We use the optimal power retention coefficient to obtain the optimal pricing so that the buyer’s utility function can reach the optimal value, and obtain the trace of the largest Fisher information matrix, that is, the largest amount of information. As defined in [8, 18], the *SPEB* is derived from the *EFIM*. We use *SPEB* to evaluate the performance of this algorithm, which is related to the information matrix. The smaller the *SPEB*, the higher the positioning accuracy. The information matrix consists of two parts, one is the initial position information obtained from the anchor node, which is related to the number of anchor nodes and the distance from the anchor node this is a constant, we do not consider this problem when optimizing. The other one is the location information obtained from other agent nodes, which is related to the power of other agent nodes to transmit location information and the location information of other agent nodes, as well as the number of optimal agent nodes to select. We give the performance evaluation criteria for collaborative positioning systems as shown below. In addition, the evaluation criteria can also be measured by mean squared error (MSE), the positioning accuracy is measured by the MSE of the agents’ estimates can be denoted by the formula $\left( 23\right)$.

22 $$\begin{aligned} SPEB&=\rho \left( P\right) =tr\left\{ \left[ J^{-1}_{P_k}\right] _{2*2} \right\} \nonumber \\&=\frac{\sum _{l=1}^{N_c}tr\left\{ \left[ J^0_{k}+\sum _{l=1,l\ne {k}}^{N_c}P_{lk}\zeta _{lk}q_{lk}q^T_{lk}\right] \right\} }{N_c} \end{aligned}$$

23 $$\begin{aligned} MSE=\frac{\sum _{k=1}^{N_{c}}\left( \left\| Q_{_{k}} -\tilde{Q_{k}}\right\| ^{2} \right) }{N_{c}} \end{aligned}$$

We use this formula $\left( 22\right)$ to calculate the average *SPEB* of the collaborative positioning system, which is a measure of positioning accuracy that best reflects the positioning performance of the system.

## Numerical results

In this section, the performance of the Cooperative positioning joint node selection and power allocation algorithm based on bidding auction is evaluated through numerical studies. Specifically, we evaluate *SPEB* and average power consumption with different positioning algorithms of the vehicle’s cooperative localization in *VANET*.

### Simulation parameters

To facilitate the expression of our proposed bidding auction-based collaborative localization node selection and power allocation algorithm for vehicular networks, we simplify this algorithm as a BACL algorithm. We verify the performance strengths and weaknesses of our algorithm based on the *SPEB* of the cooperative localization system. In the case of optimal power reservation rate, we set the simulation parameters based on several experiments as follows. We set the area as a $40m*40m$ square area and the other parameters as shown in Table 1.

**Table 1:** Table caption

$N_b$	4	The quantity of base station
$N_c$	20	The quantity of agent vehicle
$P_{s}$	1w	The direct unit transmission power
$\sigma _{lk}$	-100 dbm	The noise power of agent vehicle
$E_{all}$	1000	The initial energy
$C_{all}$	100	The initial currency

**Fig. 4:**
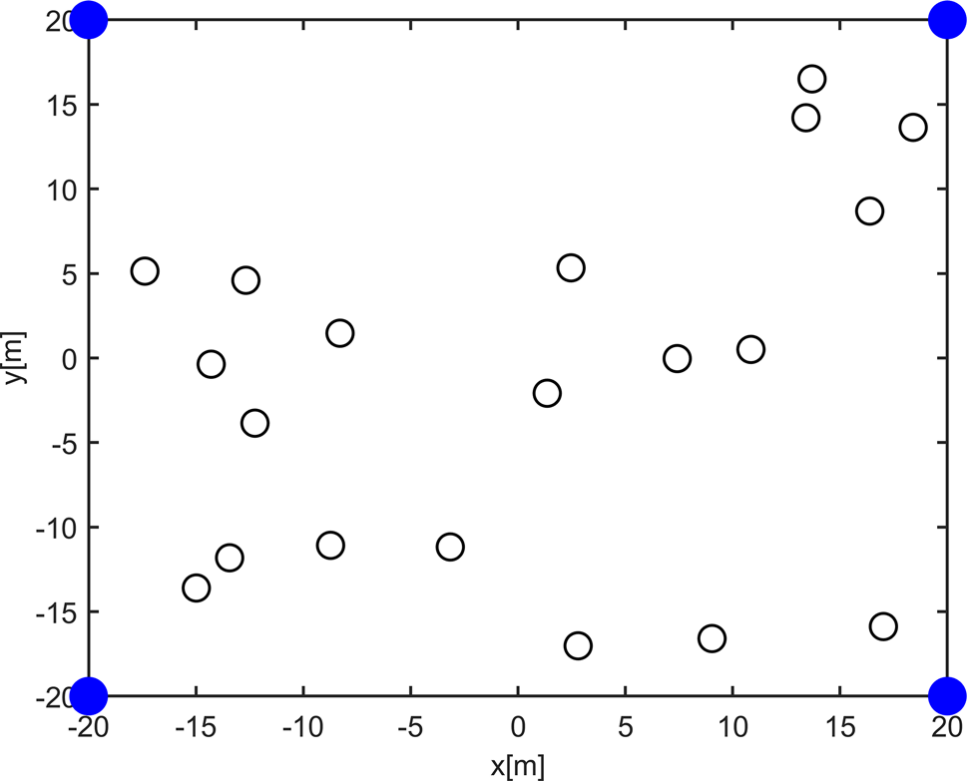
*VANET* Cooperative Positioning System Scene Map

In this paper, we model the proposed scenario and solve the formulated non-convex optimization problem using bidding auction and compare the performance with other positioning algorithms. In positioning algorithms based on bidding auction, we set the parameters of the anchor node, which is the base station in the underground parking lot, to a fixed value $N_b=4$ and the number of agent nodes to $N_C=20$ . We simplified the vehicle location scenario of the underground parking lot to Fig. 4. The simulation effect of the VANET collaborative positioning system scenario is shown in Fig. 4 below. On the one hand, it makes our proposed algorithm more universal, and on the other hand, it is more convenient for us to randomly select vehicles to participate in collaborative positioning, considering the dynamic analysis of the impact of the vehicle on the entire positioning system, further reflecting the strong generalization ability. The algorithm we proposed is tested in this figure. We use the traditional channel loss model for ranging [4], as shown in Formula $\left( 24\right)$. The direct unit transmission power is $P_S=1$, and the transmission noise $V_{lk}$ is modeled as a Gaussian random variable $\sigma _{lk}$ with variance and zero mean, which satisfies $V_{lk}\sim \mathbb {N}\left( 0,\sigma _{lk}\right)$. We set the simulation round of the auction to 100 and observe the convergence of the *SPEB* for different power allocation algorithms of the cooperative localization system. To facilitate the verification of the algorithm proposed in this paper, we assume that all agent nodes have the same initial energy $E_{all}=1000$ and the same initial currency $C_{rest}=100$.

24 $$\begin{aligned} P_{RK}=P_{Tl}-L_0-10\alpha \log _{10}{\left( \frac{d_{lk}}{d_0} \right) } +v_{lk} \end{aligned}$$

**Fig. 5:**
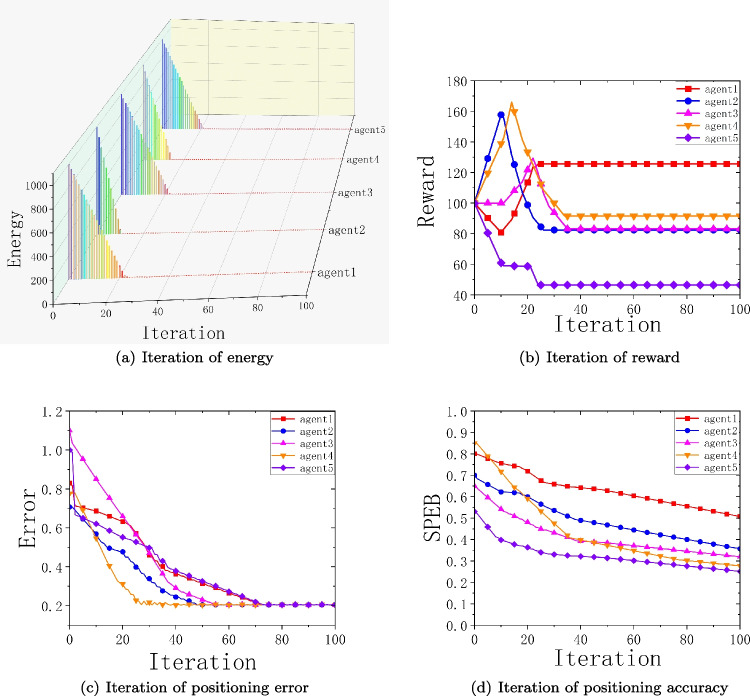
Convergence of Energy, Currency, Positioning error and *SPEB* of Different Agents

### Performance of BACL algorithm

We observe the convergence of energy consumption, virtual currency, localization error, and localization accuracy for each agent node using a bidding auction algorithm. We randomly select 5 agent nodes from the 20 agents to observe their convergence status. Fig. 5(a) shows the energy consumption of different agents with the same initial energy of each agent; Fig. 5(b) shows the situation of different agents getting rewards with the same initial currency of each agent; Fig. 5(c) shows the convergence of localization error of different agents; Fig. 5(d) shows the convergence of localization accuracy of different agents. To facilitate the reader to see the energy consumption more clearly, we use a three-dimensional view to show the energy consumption of the agent nodes. It is obvious from Fig. 5(a) that agent 2 and agent 4 have the fastest energy consumption, which can reflect that agent 2 and agent 4 are frequently selected to participate in collaborative positioning, and the virtual currency obtained will be increased first. From Fig. 5(b), we can see that the virtual currency obtained by agents 2 and 4 increases and then decreases, and when they help other agent nodes to actively participate in collaborative positioning, they can obtain the corresponding rewards. These rewards can be used to buy location information from other nodes to improve their positioning accuracy. From Fig. 5(c), we can see that the localization errors of both agent 2 and agent 4 are relatively low and converge relatively fast. Observing the localization accuracy of agent 2 and agent 4 from Fig. 5(d), it is clear that the localization accuracy is not the highest among the five randomly selected agent nodes. This is because we do not just consider the initial localization error of the node alone when choosing whether it is suitable to participate in collaborative localization of the target node, but we also need to consider other factors such as the distance of the node from the target node, the remaining energy of the node, etc. The other agent nodes are analyzed similarly to agent 2 and agent 4.

**Fig. 6:**
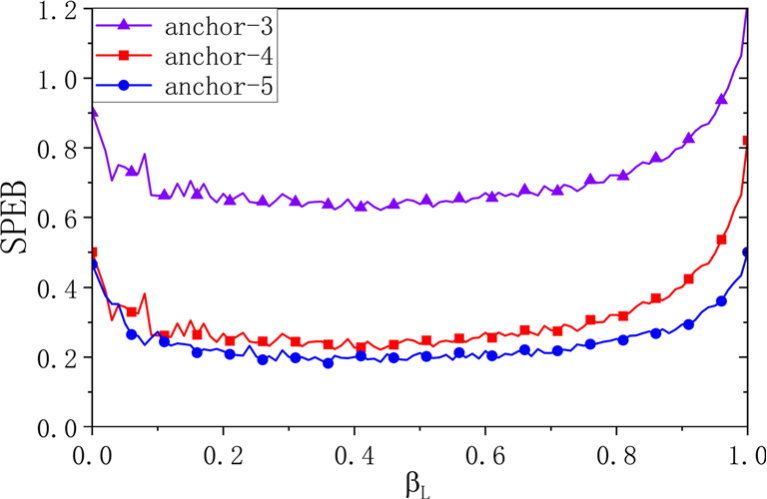
Variation of positioning accuracy with power retention for different anchor node cases

In this section, we focus on the scenario when the global information (such as channel quality, and position estimates) is available to all the agents, which thus can fully coordinate with each other. This is of particular interest in a large network. We proposed BACL algorithm can reduce the cost (objective value) for at least one agent but not increase the cost of any other agents.

### Performance evaluation and result compartion

To verify the above problem of taking the power retention rate that allows sellers to achieve the optimal utility function, we set the power retention rate as the independent variable and observe the positioning accuracy of the cooperative positioning system under different power retention rates, and the simulation results are shown in Fig. 6.

From the simulation results, it is obvious that the more anchor nodes there are, the higher the positioning accuracy of the system, but when the number of anchor nodes reaches a certain level, the improvement of the positioning accuracy of its collaborative positioning system is not obvious. When the power reservation rate is 0, the agent nodes participate in collaborative localization at full power, and when the power reservation rate is 1, the agent nodes do not participate in localization also known as non-collaborative localization. The localization accuracy is higher when the power retention is 0 than when the power retention is 1, i.e., full power collaboration is more accurate than non-collaboration. As the power retention rate increases, the agent nodes start to use part of their power for the location accuracy of the location itself, and part of it is involved in cooperative localization, so the localization accuracy starts to decrease. The *SPEB* of the cooperative localization system based on the bidding auction algorithm reaches an optimal state when the power retention rate is about 0.5. After that, the power retention continues to increase, which indicates that the power used for cooperative positioning starts to decrease, so the positioning accuracy starts to decrease. When the power retention rate is 1, at this time all nodes are selfish and are not help other nodes to improve the positioning accuracy involved in cooperative positioning, so the positioning of the whole system enters a non-cooperative state with the lowest positioning accuracy.

**Fig. 7:**
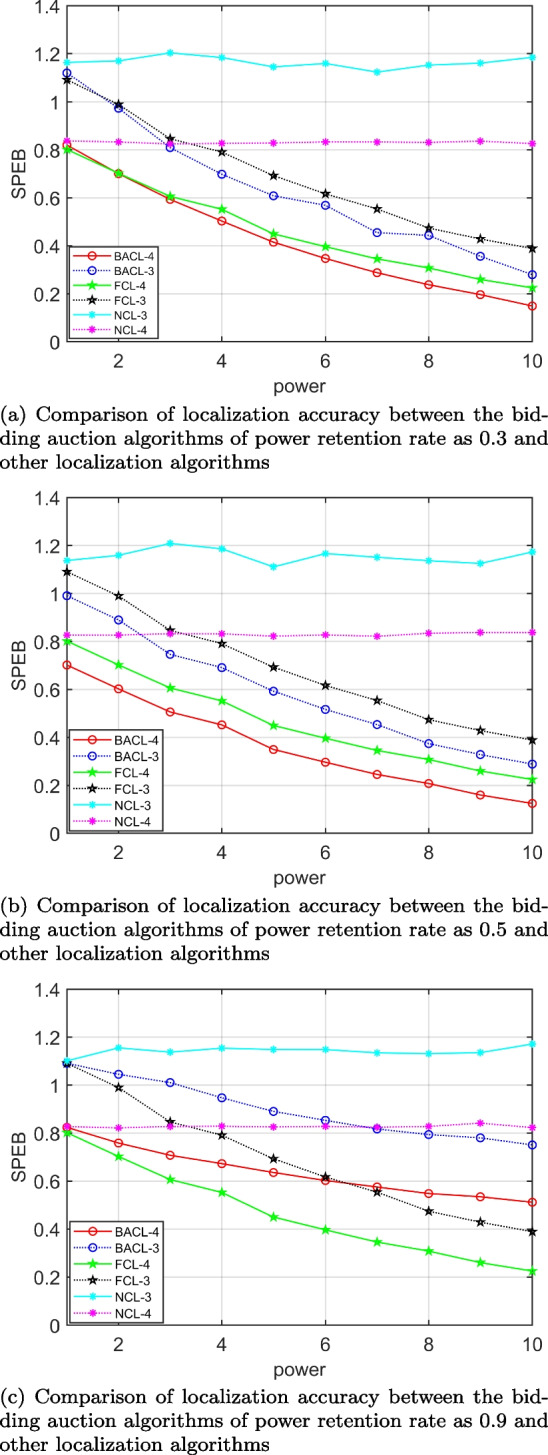
Variation of Different Cooperative Localization Algorithms with Agent Node Transmit Power: (a) Comparison of localization accuracy between the bidding auction algorithms of power retention rate as 0.3 and other localization algorithms; (b) Comparison of localization accuracy between the bidding auction algorithms of power retention rate as 0.5 and other localization algorithms; (c) Comparison of localization accuracy between the bidding auction algorithms of power retention rate as 0.9 and other localization algorithms

**Fig. 8:**
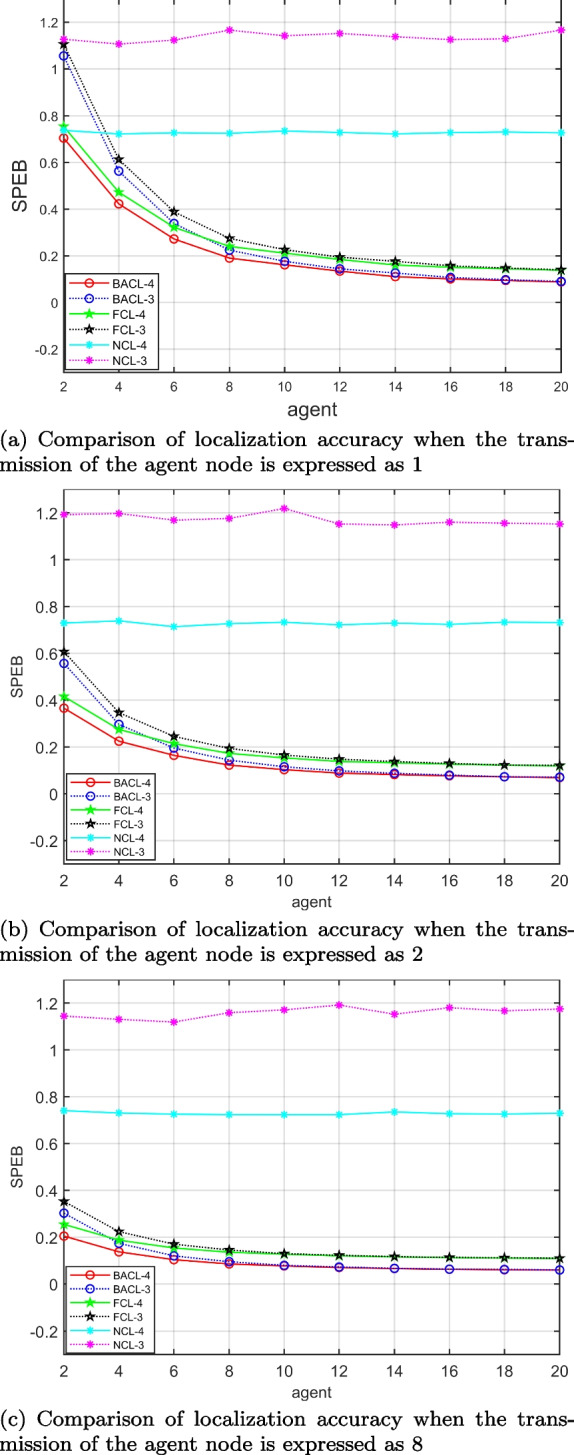
Variation of Different Cooperative Localization Algorithms with the Number of Agent Nodes: (a) Comparison of localization accuracy when the transmission of the agent node is expressed as 1; (b) Comparison of localization accuracy when the transmission of the agent node is expressed as 2; (c) Comparison of localization accuracy when the transmission of the agent node is expressed as 8

To test the performance advantages and disadvantages of our proposed scheme, we compare the BACL algorithm with the following strategies: *NCL* algorithm and *FPCL* algorithm. In the *NCL* algorithm, the position information of each agent node can only be obtained from the fixed anchor node without cooperative localization with its agent nodes, and the obtained position information is less and the positioning error is high. In the *FPCL* algorithm, agent nodes help other agent nodes to perform cooperative localization without reservation, which leads to too fast energy consumption. Although cooperative localization is performed with other agent nodes, their initial location information cannot be maintained stably, so the localization accuracy is not ideal, but the algorithm has higher localization accuracy than the *NCL* algorithm. The algorithm proposed in this paper, the *BACL* algorithm for buyers select the appropriate agent nodes to participate in their cooperative localization based on their location information, and sellers allocate the power of participating target nodes based on their EFIM, the remaining energy. We observe the system localization accuracy of different algorithms as the power increases and the cooperative agent nodes increase, respectively, and the simulation results are shown in Figs. 7 and 8.

Figure 7 shows the relationship between the *SPEB* of the average estimated position of all agents of the different power retention rate of the *BACL* algorithm compared to other localization algorithms and the transmitting power of the agent nodes. We set different power retention rates in the auction algorithm, 0.3, 0.5, and 0.9 respectively, and compare them with the FPCL and NCL algorithms. The simulation results are shown in Figs. 7(a), (b), and (c). Firstly, it is obvious that when the power retention rate is 0.5, the location effect of the auction algorithm is the best, which just confirms the previous simulation results that vary with the power retention rate. Secend, the SPEB of all cooperative schemes decreases as the transmit power of the agent nodes increases compared to the non-cooperative schemes. Finally, the *SPEB* is lower when the anchor node is 4 than when the anchor node is 3. That is, the SPEB of the system with different algorithms is lower when there are more anchor nodes, and its localization accuracy is higher.

Figure 8 shows the relationship between the *SPEB* of the average estimated position of all agents under different algorithms and the agent node when the transmission power of a single agent node is unchanged. We set different transmit power for a single proxy node, namely 1, 2, and 8, and observe the relationship between the system positioning accuracy and the number of participating nodes under different conditions. Obviously, the higher the transmission power, the higher the initial positioning accuracy. First of all, compared with non-cooperative schemes, the *SPEB* of all cooperative schemes decreases as the number of agent nodes participating in the cooperation increases. Second, the average *SPEB* of all localization algorithms when the anchor node is 4 is lower than that of the localization system when the anchor node is 3, it indicates that the more anchor nodes, the higher the positioning accuracy. Finally, the decrease is greater when the number of agent nodes involved in cooperation is 4 than when the number of agent nodes is 8, which indicates that it is not better to have a higher number of agent nodes involved in cooperation. As the number of agent nodes continues to increase, the *SPEB* of the cooperative localization system also does not converge to zero but rather converges to some positive value. It indicates that auction fatigue may occur, and we should select the appropriate agent nodes to participate in cooperative localization in practical applications.

Through the given experiments, it can be known that the proposed incentive scheme can achieve the largest positioning accuracy and the lowest power consumption, compared with other existing algorithms when the number of agent nodes and transmit power is changed. Due to these results, it can be concluded that our proposal can outperform other existing algorithms.

## Conclusion

In this paper, a cooperative localization algorithm based on a bidding auction for agent vehicles is proposed to solve the game-theoretic equilibrium problem of localization accuracy and resource consumption. To obtain optimal localization accuracy and resource allocation schemes, we formulate a BACL algorithm combining cooperative localization and bidding auction to solve a game equilibrium problem. In addition, to improve localization accuracy and reduce resource consumption, node selection incentives of the buyer and measurement power allocation strategies of the seller are adopted. Finally, we demonstrate through experiment that the proposed BACL algorithm performs excellently in terms of localization accuracy and resource consumption respectively, and can dynamically select qualified agent nodes and allocate appropriate power to transmit location information. The proposed BACL algorithm improves the positioning accuracy compared with the FPCL and NCL algorithms by 10% and 65% respectively. Meanwhile, compared with the FPCL algorithm, the proposed algorithm reduced resource consumption by 50%.

The limitation of this paper is that we only consider the problem of vehicle positioning accuracy of the static cooperative positioning system, but we neglect the dynamic analysis of the impact of joining and leaving the vehicle on the entire positioning system. For future work, we are considering extending our solution and dynamic analysis of the impact of joining and leaving the vehicle on the entire positioning system. Another perspective of this work would be to propose a cooperative game model for the vehicle positioning issue. We believe such a model would help reach better results in positioning accuracy and power loss.

## Data Availability

The manuscript does not contain any specific data set
